# Serum matrix metalloproteinase 3 levels are associated with an effect of iguratimod as add-on therapy to biological DMARDs in patients with rheumatoid arthritis

**DOI:** 10.1371/journal.pone.0202601

**Published:** 2018-08-23

**Authors:** Nao Tokai, Shuzo Yoshida, Takuya Kotani, Ayaka Yoshikawa, Yuko Kimura, Youhei Fujiki, Yoko Matsumura, Tohru Takeuchi, Shigeki Makino, Shigeki Arawaka

**Affiliations:** 1 Department of Internal Medicine (IV), Osaka Medical College, Takatsuki, Osaka, Japan; 2 Department of Internal Medicine, Arisawa General Hospital, Hirakata, Osaka, Japan; 3 Central Laboratory, Osaka Medical College, Takatsuki, Osaka, Japan; Charles P. Darby Children's Research Institute, UNITED STATES

## Abstract

**Objective:**

The aim of this study was to clarify whether serum matrix metalloproteinase 3 (MMP-3) levels are associated with an effect of iguratimod as add-on therapy to biological DMARDs (bDMARDs) in patients with rheumatoid arthritis (RA).

**Methods:**

Forty three patients with RA were treated with iguratimod as add-on therapy to bDMARDs. They were classified into remission and non-remission groups at 24 weeks of iguratimod therapy. Remission was defined as a state with a disease activity score (DAS) <2.6 in 28 joints (termed DAS remission) and total power Doppler ultrasound (US) score <3 (termed US remission). The serum MMP-3 levels at baseline and at 12 weeks were compared between these two groups.

**Results:**

There were no significant differences in the serum MMP-3 levels at baseline between the DAS and US remission groups and the non-remission group. The serum MMP-3 levels at 12 weeks in the US remission group were significantly lower than those in the non-remission group. The ratios of the serum MMP-3 levels at baseline to those at 12 weeks in both the DAS and US remission groups were significantly lower than those in the non-remission group. An MMP-3 ratio <0.86 was determined as the cut-off value to predict US remission at 24 weeks.

**Conclusion:**

Our findings suggest that the ratios of the serum MMP-3 levels at baseline to those at 12 weeks could be used to predict remission in RA patients who are administered iguratimod as an add-on to bDMARDs.

## Introduction

The goal of rheumatoid arthritis (RA) therapy is to achieve remission. Patients with RA are treated with prednisolone, conventional synthetic disease-modifying antirheumatic drugs (csDMARDs), targeted synthetic DMARDs, and biological DMARDs (bDMARDs) as monotherapy or combination therapy. If the patient shows no improvement by 3 months after the start of therapeutic intervention or the patient is unable to achieve remission by 6 months, we usually consider adjunctive therapy [1].

As the indicators of disease activity of RA, C-reactive protein (CRP), erythrocyte sedimentation rate (ESR), serum matrix metalloproteinase 3 (MMP-3), disease activity score in 28 joints (DAS28) CRP, DAS28ESR, simplified disease activity index (SDAI), and clinical disease activity index (CDAI) have been proposed. However, CRP levels do not exactly reflect the disease activity in patients with RA taking bDMARDs [2]. In addition, the ESR values are increased by anemia and hypergammaglobulinemia [3, 4]. It is thus necessary to identify useful indicators to predict the disease activity of RA.

Matrix metalloproteinases (MMPs) are secreted by chondrocytes and synovial membrane cells activated by inflammatory cytokines and closely involved in cartilage destruction. In addition to the cartilage destruction action, MMP-3 activates other MMPs including MMP-2 and MMP-9, being the most important cause of cartilage degradation [5]. Synovial fluid of RA patients contains abundant MMP-3 and core protein of proteoglycan is cut at the MMP-3-sensitive region in synovial fluid [6]. Moreover, synovial membrane with RA excessively expresses MMP-3, suggesting that MMP-3 is the key protease of articular cartilage destruction in RA.

The serum MMP-3 levels are a direct indicator of synovitis related to the disease activity of RA [7], but they are affected by other factors, such as sex difference, renal dysfunction, and corticosteroid therapy [8].

We previously reported that iguratimod improved DAS28ESR and the total power Doppler (PD) score on ultrasound (US) examination when iguratimod was added to patients resistant to bDMARDs [9]. In this study, we investigated whether the serum MMP-3 levels are a useful biomarker for predicting the therapeutic effect of iguratimod as adjunct therapy to bDMARDs in RA patients.

## Subjects and methods

### Patients

The subjects were 50 patients with RA who were treated in our hospital from January 2015 to September 2016 and showed resistance to 24-week or longer periods of bDMARDs treatment. This was defined as the state of 2.6<DAS28ESR<5.1 or total PD score ≥2 in at least one of the 28 joints on US examination. All patients met the 1987 ACR criteria [10] or 2010 ACR/EULAR classification criteria for RA [11]. Iguratimod was further administered to these patients at a dose of 25 mg/day for the first four weeks followed by an increase to 50 mg/day according to physician discretion. The serum MMP-3 levels in 47 patients were measured at baseline and at 12 weeks after the addition of iguratimod to bDMARDs treatment. Of these 47 patients, five patients were excluded from this study because erythema and infection had appeared in three patients within 24 weeks, the serum MMP-3 levels were lower than normal range at baseline in one patient, and US examinations were not performed in one patient. We analyzed DAS remission in 43 patients and US remission in 42 patients.

This study was conducted in accordance with the Declaration of Helsinki. The aim and protocol of this study were approved by the Ethics Committee of the Osaka Medical College (no. 1637). Written informed consent was obtained from all included patients.

### Assessment of serum MMP-3

The serum MMP-3 levels were measured at the beginning and 12 weeks after starting iguratimod by latex immunoassay (Panaclear MMP-3 ‘Latex’; Sekisui Medical Company Limited, Tokyo, Japan). The normal range of MMP-3 is 36.9 to 121.0 ng/mL in men and 17.3 to 59.7 ng/mL in women. The serum MMP-3 levels are affected by sex difference, renal function, and intake of corticosteroids. We calculated the serum MMP-3 ratio as that of the MMP-3 level at 12 weeks to that at baseline.

### Assessment of efficacy of iguratimod

The disease activity of RA was assessed by examining CRP, ESR, the number of tender and swollen joints, patient’s global assessment, evaluator’s global assessment, DAS28ESR, CDAI, and US (via an Aplio 300 with PLT-1204BT transducer, TOSHIBA Medical Systems Corporation, Tochigi, Japan) at baseline and at 12 and 24 weeks. Clinical assessment was performed by two different rheumatologists blinded to the US findings [9].

According to the values of DAS28ESR at 24 weeks, the patients were divided into the DAS remission group (DAS28ESR <2.6) and the DAS non-remission group (DAS28ESR ≥2.6). Similarly, according to the results of US examination, the patients were divided into the US remission group (total PD score <3) and the US non-remission group (total PD score ≥3) [12]. The MMP-3 ratio was compared between these groups. We also determined the optimal cutoff values of the MMP-3 ratio.

### Statistical analysis

Efficacy variables were calculated using the last observation carried forward method. Continuous variables were compared between groups and between each observation point using the Wilcoxon signed-rank test. Nominal variables were compared between groups using Fisher’s exact test. Receiver operating characteristic (ROC) curves were used to examine the optimal cutoff value of the MMP-3 ratio. *P* values <0.05 were considered statistically significant. Statistical analysis was performed using JMP software version 12 (SAS Institute Inc., Cary, NC, USA).

## Results

### Characteristics of the subjects

Characteristics of the subjects at baseline are shown in Table 1. The median age was 67 (60–74) years, disease duration was 7.0 (2–17) years, and 86.0% of the subjects were women. The median DAS28ESR was 3.51 (3.04–4.1), the serum MMP-3 level was 89.7 (50.6–163.1) ng/mL, and the total PD score was 8.0 (5.0–10.0). The ratio of patients using prednisolone was 53.5%, and the median methotrexate dose was 8.0 (4.0–10.0) mg/week. The number of patients using bDMARDs including overlapping use was 43. Etanercept was used in 10, adalimumab in 6, golimumab in 8, certolizumab pegol in 1, tocilizumab in 5, and abatacept in 13 patients. The dose of IGU was 25 mg/day in 10 and 50 mg/day in the other patients. The dose of IGU could not be increased due to digestive symptoms in 5, hepatopathy in 1, and malaise in 1, and arthralgia remitted after initiation of IGU administration in 3 and they did not request dose increase.

**Table 1:** Characteristics of subjects.

Characteristics	All patients (n = 43)
Age (years)	67.0 (60–74)
Female, number (%)	37 (86.0)
Disease duration (years)	7.0 (2–17)
Stage 1/2/3/4, number	7/16/6/14
Class I/II/III/IV, number	27/14/2/0
Rheumatoid factor (IU/mL)	56.0 (11–137)
Anti-CCP antibody (U/mL)	51.2 (11–100)
CRP (mg/dL)	0.06 (0.03–0.41)
ESR (mm/h)	11 (6–17)
Serum MMP-3 (ng/mL)	89.7 (50.6–163.1)
Serum creatinine (mg/dL)	0.65 (0.57–0.76)
DAS28ESR	3.51 (3.04–4.1)
CDAI	13.5 (10–17.4)
Total GS score	13.0 (9–18)
Total PD score	8.0 (5–10)
MTX use, number (%)	33 (76.7)
MTX dose (mg/week)	8.0 (4.0–10.0)
BUC use, number (%)	7 (16.3)
SASP use, number (%)	11 (25.6)
TAC use, number (%)	9 (20.9)
PSL use, number (%)	23 (53.5)
PSL dose (mg/day)	2.0 (0–5.0)
Biological DMARDs IFX/ETN/ADA/GLM/CZP/TCZ/ABT, number	0/10/6/8/1/5/13
TNFα inhibitors use, number (%)	25 (58.1)

Values indicate the median (interquartile range). CRP: C-reactive protein, ESR: erythrocyte sedimentation rate, MMP-3: matrix metalloproteinase 3, DAS: disease activity score, CDAI: clinical disease activity index, GS: gray scale, PD: power doppler, MTX: methotrexate, BUC: bucillamine, SASP: salazosulfapyridine, TAC: tacrolimus, PSL: prednisolone, DMARDs: disease modified anti-rheumatic drugs, IFX: infliximab, ETN: etanercept, ADA: adalimumab, GLM: golimumab, CZP: certolizumab pegol, TCZ: tocilizumab, ABT: abatacept, TNF: tumor necrosis factor.

### Comparison of clinical characteristics between the DAS and US remission groups and the non-remission groups at baseline

Clinical characteristics between the DAS and US remission groups and the non-remission groups at baseline can be compared in Table 2. The serum CRP and ESR levels were significantly lower in the DAS remission group than those in the DAS non-remission group (*P* = 0.045 and *P* = 0.0084, respectively). The serum CRP and ESR levels were also significantly lower in the US remission group than those in the US non-remission group (*P* = 0.0090 and *P* = 0.0495, respectively). DAS28ESR was significantly lower in the DAS remission group than that in the DAS non-remission group (*P* = 0.0008). The serum MMP-3 levels were not significantly different between the DAS and US remission groups and the non-remission groups.

**Table 2:** Comparison of clinical characteristics between the DAS and US remission groups and the non-remission groups at baseline.

	DAS			US		
Characteristics	Rem (n = 20)	Non-rem (n = 23)	*P*	Rem (n = 11)	Non-rem (n = 31)	*P*
Age (years)	65.0 (53.5–73.8)	68.0 (64–76)	0.34	69.0 (55–82)	67.0 (60–73)	0.43
Female, n	16	21	0.39	9	28	0.59
Disease duration (years)	6.5 (4–16.5)	9.0 (1.5–18)	0.98	6.0 (0.6–13)	8.0 (2–18)	0.32
Stage 1/2/3/4, n	6/6/3/5	1/10/3/9	–	4/4/2/1	3/12/4/12	–
Class I/II/III/IV, n	16/4/0/0	11/10/2/0	–	8/3/0/0	18/11/2/0	–
RF (IU/mL)	42.5 (7.25–116.5)	86.0 (31–168)	0.32	41.0 (7–56)	86.0 (20–149)	0.16
ACPA (U/mL)	50.1 (12.9–100)	57.7 (10.4–134)	0.90	49.0 (0–100)	57.0 (11–134)	0.69
CRP (mg/dL)	0.03 (0.02–0.17)	0.15 (0.04–0.6)	0.045*	0.03 (0.02–0.04)	0.15 (0.03–0.6)	0.0090*
ESR (mm/h)	7 (5.3–14.3)	12 (9–28)	0.0084*	7 (5–11)	12 (7–22)	0.0495*
Serum MMP-3 (ng/mL)	86.0 (51.4–152.8)	90.3 (48.6–170.5)	0.57	69.9 (38.7–128.4)	95.0 (53.9–170.5)	0.24
DAS28ESR	3.17 (2.84–3.50)	3.87 (3.08–4.19)	0.0008**	3.19 (2.83–3.81)	3.66 (3.16–4.18)	0.074
CDAI	12.0 (7.8–16.4)	14.5 (10.5–18.5)	0.086	14.0 (11.3–17)	13.5 (10–18)	0.90
Total GS score	14.0 (7.5–19.3)	11.0 (9–18)	0.76	9.0 (7–17)	14.0 (9–19)	0.29
Total PD score	6.5 (4–10.5)	9.0 (5–10)	0.31	6.0 (5–11)	8.0 (4–10)	0.37

Values indicate the median (interquartile range). DAS: disease activity score, US: ultrasound, Rem: remission, Non-rem: non-remission, RF: rheumatoid factor, ACPA: anti citrullinated peptide antibody, CRP: C-reactive protein, ESR: erythrocyte sedimentation rate, MMP-3: matrix metalloproteinase 3, CDAI: clinical disease activity index, GS: gray scale, PD: power Doppler.*P*-values were estimated using Fisher’s exact test or Wilcoxon signed-rank test.

*P <0.05

**P <0.001.

### Comparison of clinical characteristics between the DAS and US remission groups and the non-remission groups at 12 weeks

Clinical characteristics between the DAS and US remission groups and the non-remission groups at 12 weeks can be compared in Table 3. The serum CRP and ESR levels, DAS28ESR, and CDAI were significantly lower in the DAS remission group than those in the DAS non-remission group (*P* = 0.022, *P* = 0.0001, *P* <0.0001, and *P* <0.0001, respectively). The serum CRP, ESR, and MMP-3 levels, DAS28ESR, and total PD scores were significantly lower in the US remission group than those in the US non-remission group (*P* = 0.030, *P* = 0.010, *P* = 0.0078, *P* = 0.020, and *P* = 0.040, respectively).

**Table 3:** Comparison of clinical characteristics between the DAS and US remission groups and the non-remission groups at 12 weeks.

	DAS			US		
Characteristics	Rem (n = 20)	Non-rem (n = 23)	*P*	Rem (n = 11)	Non-rem (n = 31)	*P*
CRP (mg/dL)	0.04 (0.02–0.13)	0.12 (0.05–0.13)	0.022*	0.04 (0.01–0.07)	0.1 (0.03–0.53)	0.030*
ESR (mm/h)	6 (4.3–10.8)	18 (9–36)	0.0001**	6 (4–11)	14 (7–23)	0.010*
Serum MMP-3 (ng/mL)	69.2 (39.9–119.1)	102 (63.3–204)	0.064	51 (31.1–76.7)	102 (63.3–196.6)	0.0078*
DAS28ESR	2.26 (1.59–2.71)	4.11 (3.38–4.45)	<0.0001***	2.56 (1.25–3.15)	3.50 (2.22–4.43)	0.020*
CDAI	5.5 (4.1–8.3)	13.3 (9.4–17.7)	<0.0001***	7 (4–9.8)	10 (5.1–15.4)	0.10
Total GS score	14 (8–16)	11 (8–14)	0.34	9 (8–16)	12 (9–16)	0.34
Total PD score	4 (3–7)	6 (4–10)	0.18	3 (2–4)	6 (4–10)	0.040*

Values indicate the median (interquartile range).

DAS: disease activity score, US: ultrasound, Rem: remission, Non-rem: non-remission, CRP C-reactive protein, ESR erythrocyte sedimentation rate, MMP-3 matrix metalloproteinase, DAS disease activity score, CDAI clinical disease activity index, GS gray scale, PD power Doppler. P-values were estimated using Wilcoxon signed-rank test.

*P <0.05

**P <0.001

***P <0.0001.

**Fig 1:**
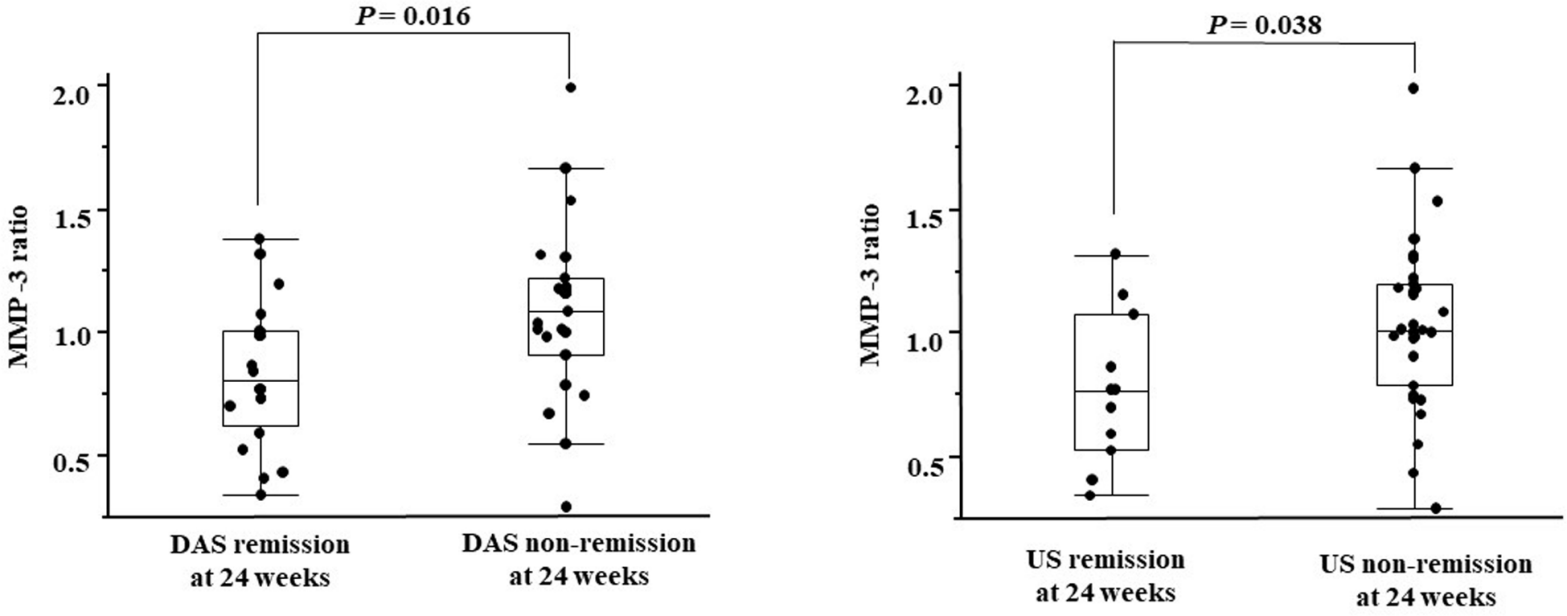
Comparison of the MMP-3 ratio between the DAS and US remission groups and the non-remission groups. MMP-3 ratio, ratio of the serum matrix metalloproteinase 3 levels at baseline to those at 12 weeks; DAS remission, disease activity score in 28 joints <2.6; US remission, total power Doppler ultrasound score <3.

A comparison of the MMP-3 ratios between the DAS and US remission groups and the non-remission group is shown in Fig 1. The median MMP-3 ratio was 0.81 (0.62–1.01) in the DAS remission group and 1.09 (0.91–1.22) in the DAS non-remission group. The MMP-3 ratio was significantly lower in the DAS remission group than that in the DAS non-remission group (*P* = 0.016). The median MMP-3 ratio was 0.77 (0.53–1.08) in the US remission group and 1.01 (0.79–1.20) in the US non-remission group. The MMP-3 ratio was significantly lower in the US remission group than that in the US non-remission group (*P* = 0.038).

### Cut-off values of the serum MMP-3 levels and the MMP-3 ratio for DAS and US remission

**Fig 2:**
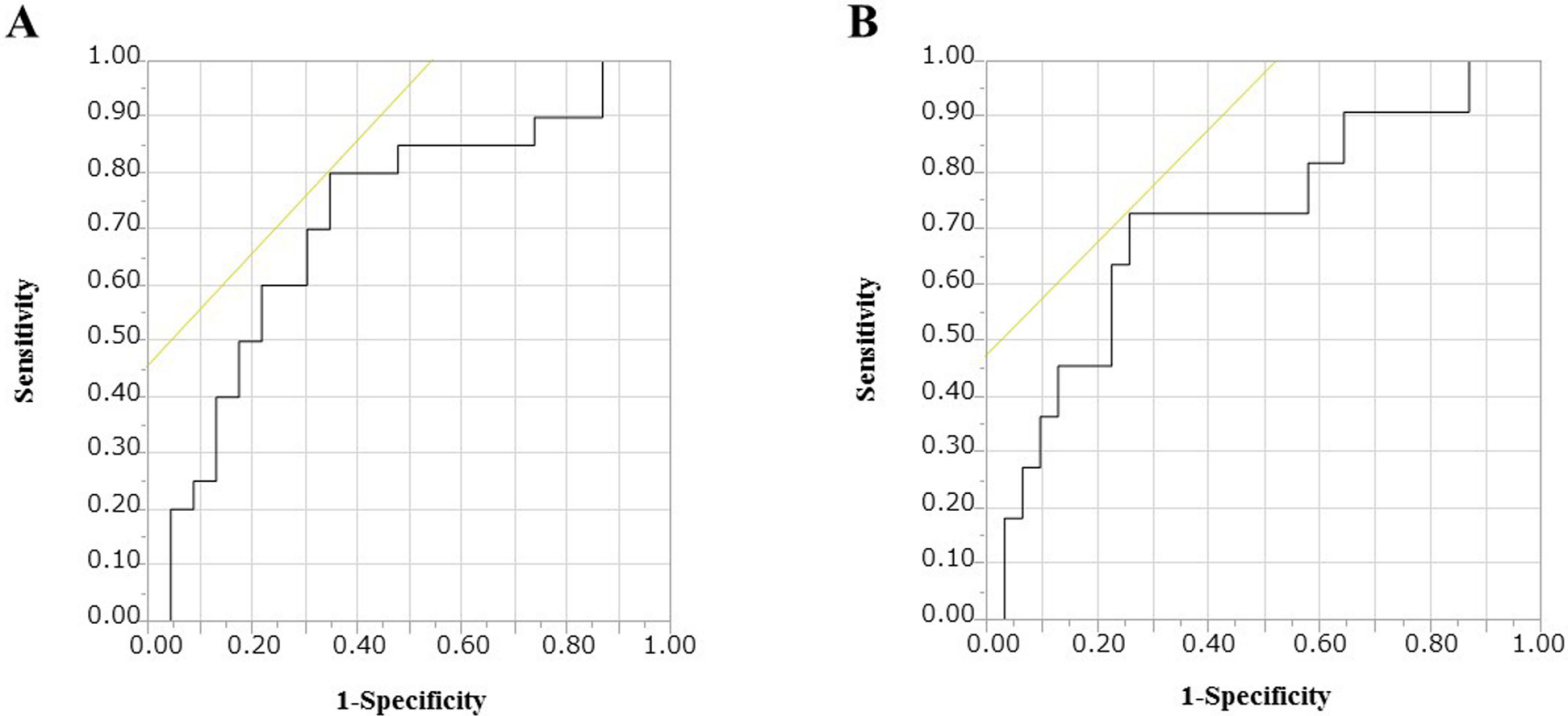
Receiver operating characteristic curves of the MMP-3 ratio for achievement of DAS and US remission at 24 weeks after addition of iguratimod. A: DAS remission, B: US remission. MMP-3 ratio, the ratio of the serum matrix metalloproteinase 3 levels at baseline to those at 12 weeks; DAS remission, disease activity score in 28 joints <2.6; US remission, total power Doppler ultrasound score <3.

To establish effective cut-off points of the serum MMP-3 levels and the MMP-3 ratio indicative of DAS and US remission, ROC curve analysis was performed on the serum MMP-3 levels and the MMP-3 ratio (Fig 2). The value for DAS remission that maximized the area under the ROC curve was 1.006 for the MMP-3 ratio (sensitivity: 80.0%, specificity: 65.2%). The values for US remission that maximized the area under the ROC curve were 76.7 for the serum MMP-3 levels (sensitivity: 81.8%, specificity: 61.4%) and 0.864 for the MMP-3 ratio (sensitivity: 72.7%, specificity: 74.3%).

The achievement ratios of DAS and US remission by the higher or lower cutoff values of the serum MMP-3 levels and the MMP-3 ratio are shown in Table 4. The achievement ratio of DAS remission of the group with an MMP-3 ratio ≤1.006 was significantly higher than that of the group with an MMP-3 ratio >1.006 (*P* = 0.014). The achievement ratio of US remission of the group with an MMP-3 ratio ≤0.864 was significantly higher than that of the group with an MMP-3 ratio >0.864 (*P* = 0.011).

**Table 4:** Cutoff values of the serum MMP-3 levels at 12 weeks for the US remission and the MMP-3 ratio for the DAS and US remission.

	Cutoff	AUC, %	DAS remission		*P*	US remission		*P*	Se, %	Sp, %	PPV, %	NPV, %	PLR
			(+)	(-)		(+)	(-)						
			n = 20	n = 23		n = 11	n = 31						
Serum MMP-3 (ng/mL)	≤76.7	77.3	–	–	–	8	12	0.081	81.8	61.4	40	86.4	2.11
MMP-3 ratio	≤1.006	71.5	15	8	0.014*	–	–	–	80	65.2	65.2	75	2.3
	≤0.864	71.3	–	–	–	8	8	0.011*	72.7	74.3	50	88.5	2.82

MMP-3: matrix metalloproteinase 3, MMP-3 ratio: the ratio of the serum matrix metalloproteinase 3 levels at baseline to those at 12 weeks, US: ultrasound, DAS: disease activity score, AUC: area under the curve, Se: sensitivity, Sp: specificity, PPV: positive predictive value, NPV: negative predictive value, PLR: positive likelihood ratio. Receiver operating characteristic curves were generated to determine the optimal cutoff value of the serum MMP-3 levels and the MMP-3 ratio. P-values were estimated using Fisher’s exact test.

*P <0.05.

## Discussion

The present study showed that the MMP-3 ratio may be useful in predicting the response of bDMARDs-treated RA patients to iguratimod add-on therapy. The probability of achieving remission was significantly higher in patients with an MMP-3 ratio ≤0.864.

Disease activity of RA is evaluated by the factors, such as CRP, ESR, and DAS28 and by US imaging of joints. DAS28, incorporating indices including the swollen joint count, tender joint count, and patient’s global health evaluation based on the visual analog scale, is the gold standard for the evaluation of disease activity. However, the swollen joint count and tender joint count in the DAS28 may show interrater variation. US imaging of joints is a very effective method of evaluating the disease state of RA, but the findings may also vary among examiners [13]. The establishment of an objective biomarker that can compensate for the defects in disease activity assessment by the DAS28 and US is awaited. CRP is widely used as marker of inflammation. However, the CRP level is low in patients using biological preparations even when the disease remains active, and so it may not accurately reflect the disease activity [9, 14, 15]. In the present study, the baseline CRP level was in the normal range in 60.5% of the patients using bDMARDs. These results also support the presence of this problem with CRP.

The MMP-3 has been reported to directly reflect synovitis and is useful as an index of the activity of RA. Hattori et al. evaluated 1321 RA patients and reported that the serum MMP-3 levels were more accurate than the serum CRP levels for assessing the likelihood of achieving SDAI remission and normal physical function [16]. Kanbe et al. evaluated 47 RA patients with high disease activity for 3 months or longer despite the use of csDMARDs or bDMARDs. They also examined predictors of treatment effectiveness by adding or changing to golimumab. They found the baseline serum MMP-3 levels to be significantly associated with DAS28CRP after 24 weeks [17]. Shiozawa et al. studied 161 RA patients treated with low-dose methotrexate for 3 years or longer without bDMARDs who fulfilled the criteria of DAS28-ESR <4.2 and CDAI <22. In those with a baseline serum MMP-3 level <103.7 ng/mL, joint radiography revealed no progression of joint destruction [18]. These reports suggest that the serum MMP-3 correlates with disease activity indices of RA and is useful as a predictor of remission. However, Urata et al. reported that the serum MMP-3 alone did not serve as an index of the likelihood of remission in 243 RA patients [19]. The results of the present study suggest that in RA patients treated with bDMARDs, the serum MMP-3 levels measured at 12 weeks may be useful for the prediction of US remission at 24 weeks after the initiation of iguratimod add-on therapy.

The serum MMP-3 levels are known to differ between men and women and to be high in patients with reduced renal function [8]. Concomitant drugs may influence the serum MMP-3 levels and response. The serum MMP-3 levels at 12 weeks were significantly higher in the IGU 50 mg/day than IGU 25 mg/day group, but this may have been due to the influence of the fact that the baseline serum MMP-3 levels were already high in the IGU 50 mg/day group. There was no significant difference in the MMP-3 ratio between the IGU 25 and IGU 50 mg/day groups, suggesting that the IGU dosage was unlikely to have an influence on the response of MMP-3. No significant difference due to the presence or absence of concomitant MTX was noted in the serum MMP-3 levels at 12 weeks or MMP-3 ratio, and the MTX dose at 12 weeks was not correlated with the serum MMP-3 levels at 12 weeks or MMP-3 ratio.

**Fig 3:**
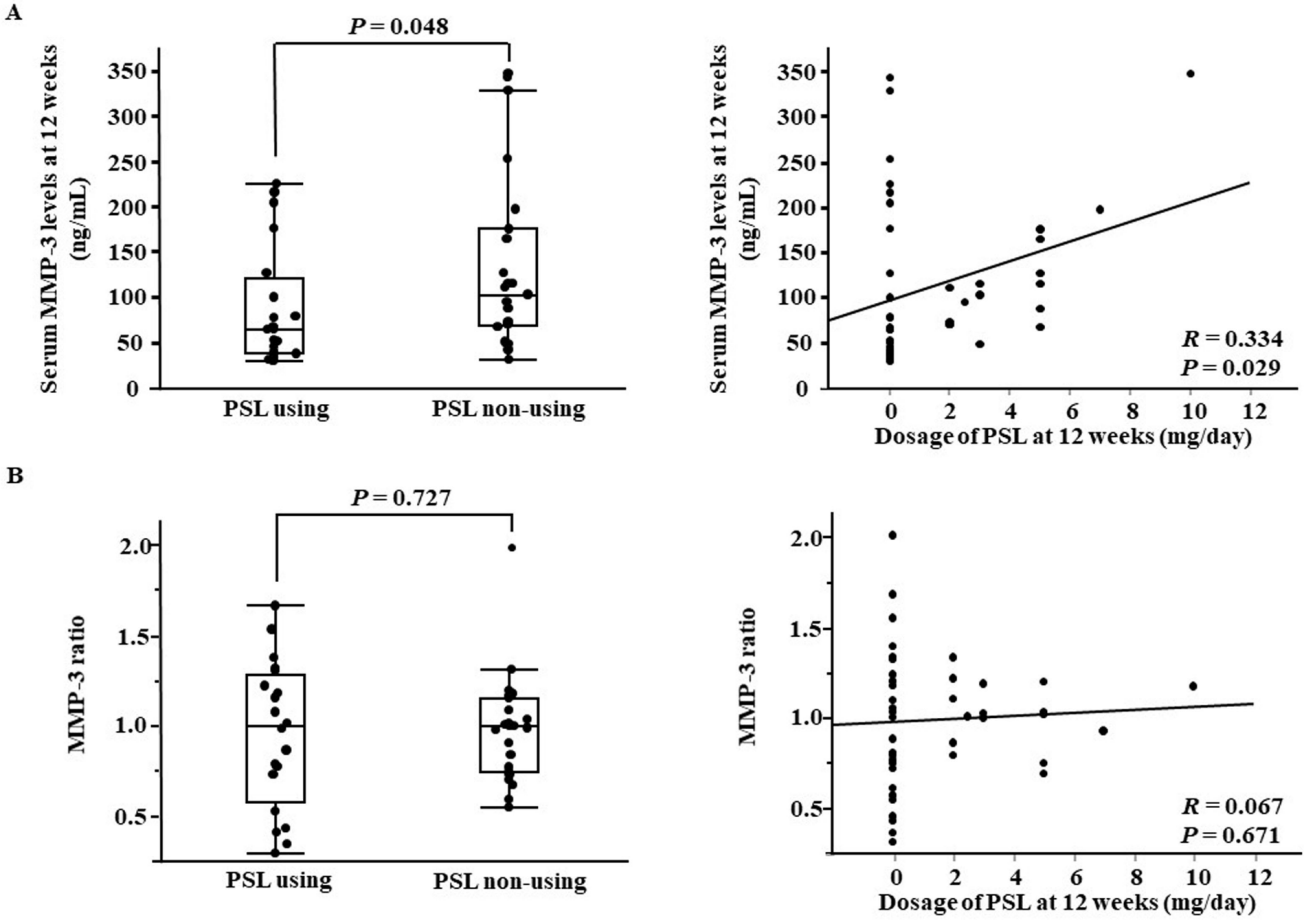
**Comparison of the serum MMP-3 levels at 12 weeks and the MMP-3 ratio between steroids using and non-using groups (A). Correlation between the serum MMP-3 levels at 12 weeks / the MMP-3 ratio and dosage of steroids (B).** MMP-3 ratio, ratio of the serum matrix metalloproteinase 3 levels at baseline to those at 12 weeks; PSL, prednisolone.

In addition, the serum MMP-3 levels are reduced rapidly by the administration of tocilizumab [20] but slowly by the administration of abatacept [21]. It is also affected by the type of bDMARD used. The number of patients in the present study was limited, and a wide variety of bDMARDs were used. Therefore, the effects of steroid or bDMARDs on the serum MMP-3 levels could not be evaluated accurately. Steroid elevates the serum MMP-3 levels [8]. Actually, in the present study, the serum MMP-3 levels at 12 weeks was higher in patients treated with concomitant steroid than in those without it and a significant positive correlation was noted between the steroid dose at 12 weeks and serum MMP-3 levels. However, no significant difference due to concomitant steroid or correlation with the steroid dose was noted in the MMP-3 ratio (Fig 3). It was suggested that the MMP-3 ratio is more useful than the serum MMP-3 levels to predict remission in patients treated with steroid.

The influence of the concomitant drugs described above as a confounding factor which may influence the serum MMP-3 levels and response could not be investigated using multivariate analysis because of the small number of patients.

The result that the baseline serum MMP-3 levels did not correlate with the therapeutic effect may have been due to the above sex difference or concomitant medications.

According to a report by Hattori et al. analyzing 114 RA patients treated with adalimumab for 52 weeks or longer, the rate of improvement of the serum MMP-3 levels after 4 weeks was significantly lower in the DAS28-CRP remission group after 52 weeks than in the non-remission group. In addition, the cut-off value for the rate of serum MMP-3 levels improvement to predict remission was 39.9% [22]. The serum MMP-3 levels and their change from the baseline value may be useful for the prediction of the disease activity of RA and the likelihood of remission. The present study showed the possibility that the MMP-3 ratio may also be useful to predict the efficacy of iguratimod add-on therapy after 24 weeks in RA patients using bDMARDs.

US remission is more favorable than DAS remission. Joint destruction progresses in some patients who have achieved DAS remission if PD persists on US [23, 24]. Therefore, if possible, US remission is desirable as the primary goal. The present study showed the possibility that US remission may be predictable by evaluating the serum MMP-3 levels and the MMP-3 ratio.

There are several limitations of this study: This was a single center study, the number of patients was small as a cohort study, and no control group was set. Moreover, there are diverse types of bDMARDs used in this study and it is necessary to investigate the efficacy of add-on IGU by the type of bDMARD. Moreover, the effects of sex differences or concomitant medications were not thoroughly evaluated, as mentioned above. In the future, it would be necessary to prospectively accumulate cases and clarify the usefulness of the serum MMP-3 for the prediction of DAS and US remission in RA patients.

## Conclusion

The ratios of the serum MMP-3 levels at baseline to those at 12 weeks may be a useful index to predict the response to iguratimod add-on therapy 24 weeks after its initiation in RA patients treated with bDMARDs. It is necessary to investigate the dose and type of add-on csDMARDs including IGU and the type of bDMARDs to which csDMARDs are added.
