# Correction: Serum matrix metalloproteinase 3 levels are associated with an effect of iguratimod as add-on therapy to biological DMARDs in patients with rheumatoid arthritis

**DOI:** 10.1371/journal.pone.0211750

**Published:** 2019-01-30

**Authors:** Nao Tokai, Shuzo Yoshida, Takuya Kotani, Ayaka Yoshikawa, Yuko Kimura, Youhei Fujiki, Yoko Matsumura, Tohru Takeuchi, Shigeki Makino, Shigeki Arawaka

There is an error in the second sentence of the Subjects and Methods section. The correct sentence is: This was defined as the state of 2.6 < DAS28ESR < 5.1 or PD score ≥ 2 in at least one of the 28 joints on US examination.

There is an error in the fourth sentence of the fifth paragraph of the Discussion section. The correct sentence is: Therefore, the effects of bDMARDs on the serum MMP-3 levels could not be evaluated accurately.

There is an error in the first sentence of the eighth paragraph of the Discussion section. The correct sentence is: According to a report by Hattori et al. analyzing 114 RA patients treated with adalimumab for 52 weeks or longer, the rate of improvement of the serum MMP-3 levels after 4 weeks was significantly higher in the DAS28-CRP remission group.

In Fig 3, the headings PSL using and PSL non-using are swapped. The left side should be PSL non-using and the right should be PSL using. Please see the corrected Fig 3 here.

**Fig 3:**
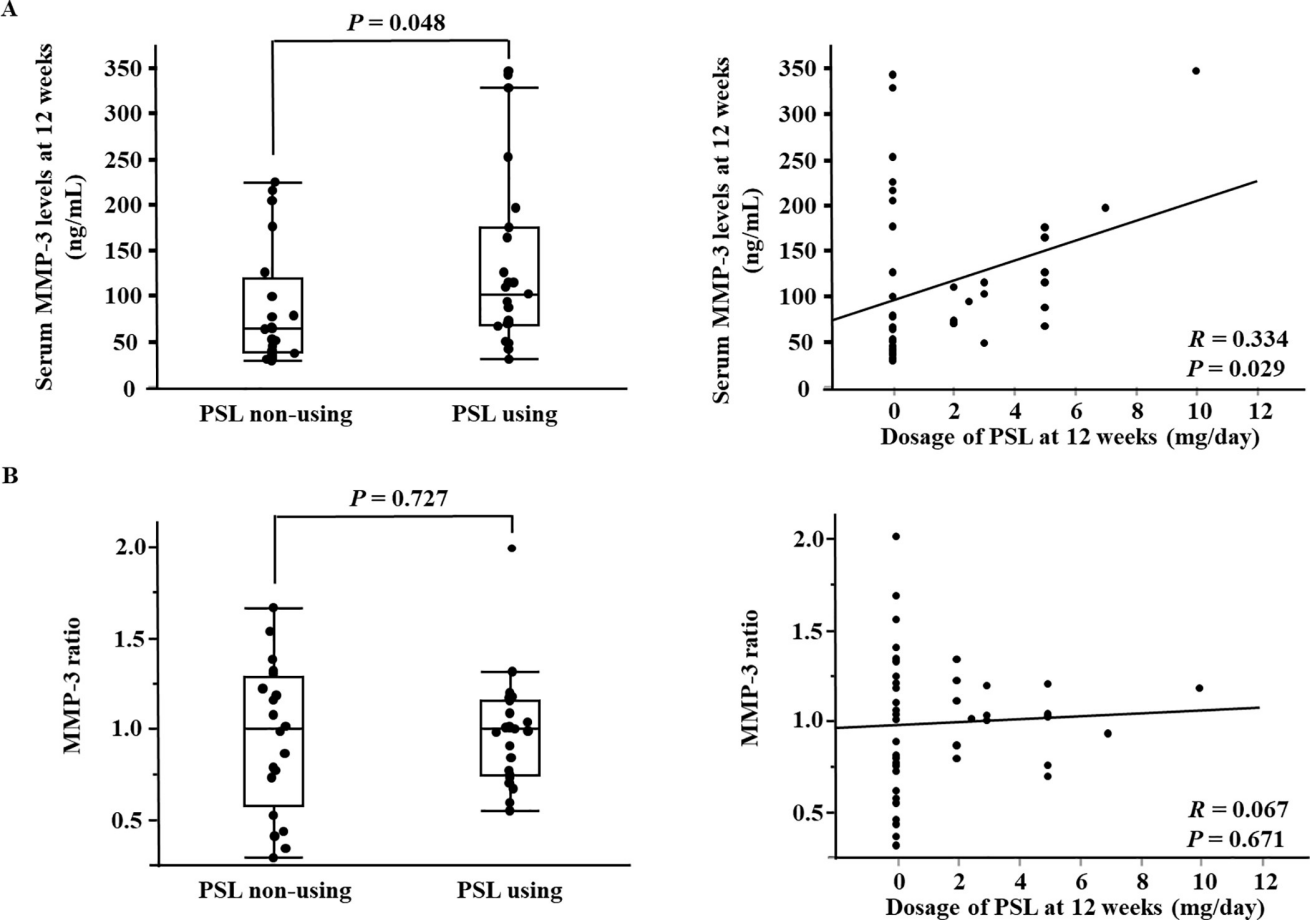
**Comparison of the serum MMP-3 levels at 12 weeks and the MMP-3 ratio between steroids using and non-using groups (A). Correlation between the serum MMP-3 levels at 12 weeks / the MMP-3 ratio and dosage of steroids (B).** MMP-3 ratio, ratio of the serum matrix metalloproteinase 3 levels at baseline to those at 12 weeks; PSL, prednisolone.
